# Neuroprotection of Anakinra on peripheral nerve neurodegeneration in single and combination protocols with TTR siRNA in a transgenic mouse model for human V30M transthyretin

**DOI:** 10.1186/1750-1172-10-S1-P13

**Published:** 2015-11-02

**Authors:** Maria João Saraiva, Paula Gonçalves, Diana Martins, Nádia Pereira Gonçalves

**Affiliations:** 1 Instituto de Inovação e Investigação em Saúde (I3S), Instituto de Biologia Molecular e Celular (IBMC), Universidade do Porto, Molecular Neurobiology Unit, 4150-180, Porto, Portugal

## Background

We previously showed the properties of interleukin-1β antagonist Anakinra on unmyelinated nerve fibers protection in a transgenic mouse model for human V30M transthyretin. In these studies, Anakinra decreased, among other markers, nerve levels of IL-1β, NF-κB and activated-caspase 3, associated with a decrease in TTR-non fibrillar deposition.

In the present work, we compared the efficacy of other compounds under human therapeutical trials for Familial Amyloidotic Polyneuropathy (FAP) in the same animal model, having 5 months of age.

## Methods

V30M transgenic mice were treated daily with subcutaneous injections of Anakinra at 25 mg/kg over 6 weeks. Age-matched controls were injected with phosphate buffer saline (PBS). TTR siRNA, at a concentration of 1 mg/kg was injected in the tail vein for 4 weeks. One intravenous injection was performed per week and animals were euthanized 48 h after the last injection. Untreated age-matched controls received vehicle intravenously. Anakinra plus TTR siRNA combination therapy was performed using the same therapeutical design. In addition, other V30M mice group was treated with Tafamidis meglumine with 3 subcutaneous injections weekly, over 6 weeks. Controls received meglumine alone. Finally, combination strategy with Doxycycline/TUDCA was also achieved in V30M mice. Animals were treated with 8 mg/kg Doxycycline daily in the drinking water and received intraperitoneal injections of 500 mg/kg TUDCA twice a week for 4 weeks. Controls were injected with intraperitoneal PBS.

After mice sacrifice, nerves were collected into a 0.1 M sodium cacodylate solution containing 1.25% glutaraldehyde and 4% paraformaldehyde for posterior fiber counting. Nerves from animals treated with Anakinra, TTR siRNA or both agents combined were also analyzed by immunohistochemistry for inflammatory and apoptotic markers, namely NF-κB transcription factor, IL-1β and FAS death receptor.

## Results

In contrast with Anakinra treatment alone, no differences in both myelinated and unmyelinated fiber density as compared with vehicle were found for the other tested compounds. However, combined Anakinra and siRNA administration resulted in increased density of unmyelinated fibers as compared to controls. Efficiency of Anakinra as a neuroprotective molecule was corroborated in sciatic nerve analyses of NF-κB, FAS death receptor and IL-1β in animals treated with a combination of Anakinra and siRNA, since these markers were found downregulated in animals receiving this combined therapy. Mice treatment with single protocols of siRNA, Tafamidis or Doxycycline/TUDCA, did not change levels of the selected biomarkers.

## Conclusion

Anakinra should be considered for its potential in single and/or combination protocols for FAP studies.

